# PDL1 And LDHA act as ceRNAs in triple negative breast cancer by regulating miR-34a

**DOI:** 10.1186/s13046-017-0593-2

**Published:** 2017-09-15

**Authors:** Xiaojia Huang, Xinhua Xie, Hua Wang, Xiangsheng Xiao, Lu Yang, Zhi Tian, Xiaofang Guo, Lijuan Zhang, Hailin Tang, Xiaoming Xie

**Affiliations:** 1 Department of Breast Oncology, Sun Yat-sen University Cancer Center, State Key Laboratory of Oncology in South China, Collaborative Innovation Center for Cancer Medicine, 651 East Dongfeng Road, Guangzhou, 510060 People’s Republic of China; 2 Department of Hematological Oncology, Sun Yat-sen University Cancer Center, State Key Laboratory of Oncology in South China, Collaborative Innovation Centre for Cancer Medicine, Guangzhou, 510060 People’s Republic of China; 3 College of Pharmacy, University of South Florida, Tampa, USA

**Keywords:** PDL1, LDHA, miR-34a, Competitive endogenous RNAs, Triple negative breast cancer

## Abstract

**Backgroud:**

The purpose of this study was to elucidate the regulation of programmed death ligand 1 (PDL1), lactate dehydrogenase A (LDHA) and miR-34a in triple negative breast cancer (TNBC) and to explore the function and mechanism of PDL1 and LDHA as competitive endogenous RNAs (ceRNAs) in TNBC via regulation of miR-34a.

**Methods:**

Western blotting, quantitative RT-PCR (qRT-PCR) and immunohistochemistry (IHC) assays were conducted to explore the expression of PDL1, LDHA and miR-34a in TNBC and correlations between them. MTS cell viability, Transwell migration, glucose consumption and lactate production assays and flow cytometry were performed and mouse xenograft models were constructed to explore the functions and regulation of the PDL1 3’UTR and LDHA 3’UTR and miR-34a in TNBC.

**Results:**

We found that PDL1 and LDHA were synchronously upregulated in TNBC cell lines and tissues. Co-expression of PDL1 and LDHA was correlated with poor outcome in TNBC. Both PDL1 and LDHA are targets of miR-34a, and the 3’UTRs of PDL1 and LDHA both have binding sites for miR-34a. The functions of PDL1 and LDHA were inhibited by miR-34a. In addition, PDL1 and LDHA acted as ceRNAs to promote the expression and function of each other through regulation of miR-34a in TNBC.

**Conclusions:**

This study provides a new theoretical basis for a novel TNBC therapeutic strategy. Simultaneously targeting PDL1 and LDHA, which would combine immunotherapy and metabolically targeted treatments, might shed some light on the treatment of breast cancer, especially TNBC.

## Background

Programmed death ligand 1 (PDL1) is highly expressed on the surface of a variety of cancer cells. It is well known that PDL1 inhibits the proliferation and function of tumor-infiltrating lymphocytes (TILs), leading to an immunosuppressive environment in cancers [1–4]. In addition, PDL1 also has important functions in cancer signaling and survival, but these are little studied. Moreover, there is still not enough evidence regarding the expression pattern and functions of PDL1 in triple negative breast cancer (TNBC).

Altered energy metabolism is common in cancer cells and has been regarded as a hallmark of cancer [5]. Oncogene-induced metabolic reprogramming fuels the growth and proliferation of cancer cells [6]. Lactate dehydrogenase A (LDHA) is a glycolytic enzyme that is essential for cancer energy metabolism, and it is highly expressed in cancer cells and correlated with poor survival [7]. Inhibition of LDHA has an anti-proliferative effect on cancer cells. However, the expression pattern and functions of LDHA in TNBC are still not clear.

It is reported that RNAs can act as competitive endogenous RNAs (ceRNAs) to co-regulate each other by competing for shared microRNAs [8, 9], and the 3′ UTR of mRNA alone is capable of eliciting ceRNA effects [10]. Recent studies have reveal that miR-34a suppresses PDL1 expression by directly targeting the 3′ UTR of PDL1 in lung cancer and acute myeloid leukemia [11, 12]. Other studies have confirmed that miR-34a targets LDHA in colorectal cancer and breast cancer [13, 14]. Both the 3’UTR of LDHA and PDL1 have the miR-34a binding site, which denotes that they could act as ceRNAs by competing for miR-34a.

To elucidate the regulation of PDL1, LDHA and miR-34a in TNBC, this study will explore the function of miR-34a as a tumor suppressor that regulates glycolysis and immunology in TNBC cells by targeting LDHA and PDL1 and examine the mechanism of PDL1 and LDHA as ceRNAs that promote proliferation and metastasis of TNBC by regulating miR-34a. Ultimately, this study will provide a new theoretical basis for a novel therapeutic strategy for TNBC.

## Methods

### Cell lines and culture

The human mammary epithelial (HME) cell line MCF-10A and TNBC cell lines MDA-MB-453, MDA-MB-468, MDA-MB-231, BT-549, HCC38 and 4 T1 were obtained from the American Type Culture Collection (ATCC, USA). The TNBC cell lines were cultured in RPMI-1640 medium (Gibco, USA) supplemented with 10% fetal bovine serum (Gibco, USA) at 37 °C in a humidified atmosphere with 5% CO_2_. All cell lines were re-authenticated by short tandem repeat DNA profiling every six months.

### Clinical samples

Tissue samples comprising 20 TNBC tissues and their corresponding paired normal adjacent tissues were immediately cut and stored in RNAlater (Ambion, USA) and subjected to quantitative real-time PCR (qRT-PCR) analysis. The tissue microarrays (TMAs) consisted of 554 cases of breast cancer tissues diagnosed in Sun Yat-sen University Cancer Center. Specimens were obtained during surgery and fixed in formalin and embedded in paraffin through standard methods. TMAs were constructed as follows. Briefly, histological slides were retrieved and reviewed, and representative tumor areas were selected for TMA construction using a Beecher microarray with 1-mm cores. The presence of carcinoma in the core was used as an inclusion criterion. We compared both hematoxylin and eosin (HE) stained and immunostained TMA sections with the corresponding full-section slides to assess the representativeness and heterogeneity of staining. This study was approved by the Ethics Committees of Sun Yat-sen University Cancer Center, and conducted in accordance with the Helsinki Declaration. Informed consent was obtained from all patients included in the study.

### Quantitative RT-PCR analysis (qRT-PCR)

Total RNA was extracted using TRIzol reagent (Invitrogen, USA) based on the manufacturer’s instructions. Reverse transcription and qRT-PCR reactions of mRNAs were performed using PrimeScript™ RT Master Mix and SYBR® Premix Ex TaqTM II (Takara, Japan). The primers for LDHA, PDL1 and β-actin were synthesized by Invitrogen: hsa-LDHA-forward, 5′-ATGGCAACTCTAAAGGATCAGC-3′; hsa-LDHA-reverse, 5′-CCAACCCCAACAACTGTAATCT-3′; mus-LDHA-forward, 5′-TGTCTCCAGCAAAGACTACTGT-3′; mus-LDHA-reverse, 5′-GACTGTACTTGACAATGTTGGGA-3′; hsa-PDL1-forward, 5′-GACATGTCAGGCTGAGGGCT-3′; hsa-PDL1-reverse, 5′-TGATTCTCAGTGTGCTGGTCACA-3′; mus-PDL1-forward, 5′-GCTCCAAAGGACTTGTACGTG-3′; mus-PDL1-reverse, 5′-TGATCTGAAGGGCAGCATTTC-3′; hsa-β-actin-forward, 5′-AGCGAGCATCCCCCAAAGTT-3′; hsa-β-actin-reverse, 5′-GGGCACGAAGGCTCATCATT-3′; mus-β-actin-forward, 5′-GTGACGTTGACATCCGTAAAGA-3′; and mus-β-actin-reverse, 5′-GCCGGACTCATCGTACTCC-3′. Reverse transcription and qRT-PCR reactions of miRNA were performed using an All in One™ miRNA qRT-PCR Detection Kit (GeneCopoeia, USA). The primers for miR-34a and U6 snRNA were purchased from GeneCopoeia. The threshold cycle (CT) value for LDHA or PDL1 was normalized against that of β-actin, while U6 snRNA was used as an internal control for miR-34a. The relative fold change was calculated by the 2-ΔΔCt method. All the qRT-PCR assays were performed with a Bio-Rad CFX96 PCR System (USA).

### Western blot

The expression levels of LDHA and PDL1 were detected by Western blotting. Total protein was extracted from cell lines and xenograft tumor tissues using RIPA lysis buffer with a proteinase inhibitor. The protein concentrations in the lysates were measured using a Protein BCA Assay Kit (Bio-Rad, USA), and 100 μg of protein mixed with 5× SDS loading buffer was loaded per lane. The proteins in the lysates were separated by 10% SDS-polyacrylamide gel electrophoresis and transferred to polyvinylidene difluoride membranes (Merck Millipore, USA). To block nonspecific binding, the membranes were incubated with 5% skim milk powder at room temperature for an hour. Then, the membranes were incubated with antibodies against LDHA (dilution 1:1000, Affinity, USA, catalog #: DF6280) and PDL1 (dilution 1:1000, Invitrogen, USA, catalog #: PA5–20343) overnight at 4 °C. A peroxidase-conjugated secondary antibody (1:3000 dilution) and ECL Western blotting detection reagents (ECL New England Biolabs, USA) were used to visualize the target proteins, which were quantified with Image Lab (Bio-Rad, USA). An anti-β-actin antibody (dilution 1:1000, Affinity, USA, catalog #: AF7018) was used as a protein loading control.

### Immunohistochemistry (IHC) analysis and scoring system

After deparaffinization and rehydration, the slides were treated with 90% methanol/3% H_2_O_2_ solution for 10 min at room temperature to block endogenous peroxidase. Then, the slides were soaked in sodium citrate buffer (10 mM sodium citrate, 0.05% Tween 20, pH 6.0) at 96 °C for 5 min for antigen retrieval. After blocking with BSA, the following antibodies were used: antibody against LDHA (dilution 1:100, Affinity, USA) and antibody against PDL1 (dilution 1:100, Invitrogen, USA). The slides were stored at 4 °C overnight and then incubated with biotinylated secondary antibody and HRP-streptavidin for 10 min each at room temperature. After DAB staining, the results were graded for intensity using a semi-quantitative histochemical scoring (H score) system. Staining intensity was scored as 0, 1+, 2+, or 3+ for negative, weak, moderate, and strong staining, respectively. Final scores were calculated as follows: (3 × % strong staining) + (2 × % moderate staining) + (1 × % weak staining), producing an H score ranging from 0 to 300. Immunoreactivity was categorized into negative expression (H score 0–99) and positive expression (H score 100–300) [15]. Scoring of IHC staining was performed by two pathologists in a blinded manner.

### Plasmid construction and establishment of stable cell lines

For overexpression of the LDHA 3′ UTR or PDL1 3′ UTR, fragments containing the binding sites for miR-34a in the 3′ UTR of LDHA or PDL1 were synthesized by GeneCopoeia and subcloned into the eukaryotic expression vector pEZ-Lv201 (GeneCopoeia, USA). The mutant vectors which the first five nucleotides complementary to miR-34a binding sites were mutated by site-directed mutagenesis (Stratagene) served as mutant controls. The constructs were verified by DNA sequencing. The vector Lv201CT (GeneCopoeia, USA) was used as a negative control.

For lentivirus packaging, a Lenti-Pac™ HIV Expression Packaging Kit (GeneCopoeia, USA) was used based on the manufacturer’s protocol. The plasmids were then transfected into 293 T cells and virus-containing medium supernatants were used to infect HCC38, MDA-MB-231 and 4 T1 cells using Lipofectamine 2000 transfection reagent (Invitrogen, USA) based on the manufacturer’s protocol. Infected cells were selected with 2 μg/mL puromycin for two weeks (Invitrogen, USA) until drug-resistant cells were obtained. Then, the stable cell lines were expanded, and forced expression was confirmed. Then, the cells were prepared for the following experiments.

### MTS cell viability assay

The cell viability was evaluated with an MTS assay kit (BestBio, China). Cells were seeded at a density of 5000 cells per well in 96-well plates and cultured at 37 °C for 24 h before transfection. The MTS assays were performed 48 h after the transfection based on the manufacturer’s instructions. The absorbance values were measured at 490 nm using a Biotek Epoch 2 microplate spectrophotometer (Biotek, USA). Triplicate independent experiments were performed, and the viability of cells was calculated as the percentage of treated cells to control cells.

### Transwell migration assay

Cells invasion capacity was determined with Transwell migration assays. Briefly, cells were seeded onto the basement membrane matrix in the insert of 24-well culture plates (EC matrix, Chemicon, USA). Then, 20% fetal bovine serum was added to the lower chamber as an attractant. After 48 h, the non-invading cells and the EC matrix were gently removed with a cotton swab. Invasive cells located on the lower side of the chamber were fixed and stained with crystal violet, imaged and counted. Triplicate independent experiments were performed.

### Mouse xenograft model

A total of 2 × 10^5^ MDA-MB-231/PDL1 cells, MDA-MB-231/LDHA cells or MDA-MB-231/vec cells were injected into the mammary fat pad of nude mice (six mice in each group). After 28 days, the mice were euthanized, and the tumors were subjected to Western blotting.

A total of 2 × 10^5^ 4 T1/PDL1 cells, 4 T1/LDHA cells or 4 T1/vec cells were injected into the mammary fat pad of immunocompetent C57BL/6 mice (five mice in each group). After 28 days, the mice were euthanized, and tumor lysates were subjected to flow cytometry.

All the animal procedures were performed in accordance with the institutional standard guidelines of Sun Yat-sen University Cancer Center.

### Flow cytometry

Isolation of cells and flow cytometry was performed as described before [11]. Briefly, freshly isolated xenograft tumor tissues were washed with ice-cold PBS and digested in PBS supplemented with 3 mg/mL dispase II and 2 mg/mL collagenase at 37 °C for an hour. Single cell suspensions were prepared by filtering the digested tissues through cell strainers with 70-μm pores. Then, erythrocytes were removed with red blood cell lysis buffer, and single-cell suspensions were used for flow cytometry staining. CD45 staining was used to distinguish immune cells from non-immune cells (tumor and other stromal cells) in tumor tissues, and the cells quantified were CD8+ T cells, CD4+ T cells, macrophages (F4–80+) and T-regulatory T cells (Tregs) (CD4 + FOXP3+). Samples were analyzed with a Gallios Flow Cytometer (Beckman-Coulter, USA), and the data were analyzed with FlowJo Software. The expression of each marker in the control group was considered the basal expression level.

### Measurement of lactate production and glucose consumption

The stable cell lines HCC38/PDL1, MDA-MB-231/PDL1, HCC38/vec and MDA-MB-231/vec were cultured, and the culture media were collected. Lactate production and glucose consumption were measured using a Lactate Colorimetric Assay Kit II (BioVision, USA) and a Glucose Colorimetric Assay Kit II (BioVision, USA), respectively. Triplicate independent experiments were performed, and the results were normalized based on total cellular protein amounts.

### Statistical analysis

Comparisons between groups were analyzed with t-tests. Paired Student’s t-tests were used to compare mRNA or miRNA levels between TNBC samples and corresponding normal tissues. Two-tailed Pearson correlation was used to examine the correlation between LDHA and PDL1 expression. Kaplan-Meier plots and log-rank tests were used for survival analysis. Unless otherwise indicated, the data are reported as the mean ± s.e.m. at a significance level of *P* < 0.05. All the statistical analyses were performed using the SPSS 19.0 statistical package (SPSS Inc., USA).

## Results

### PDL1 Is highly expressed in TNBC and correlated with a poor outcome

**Fig. 1:**
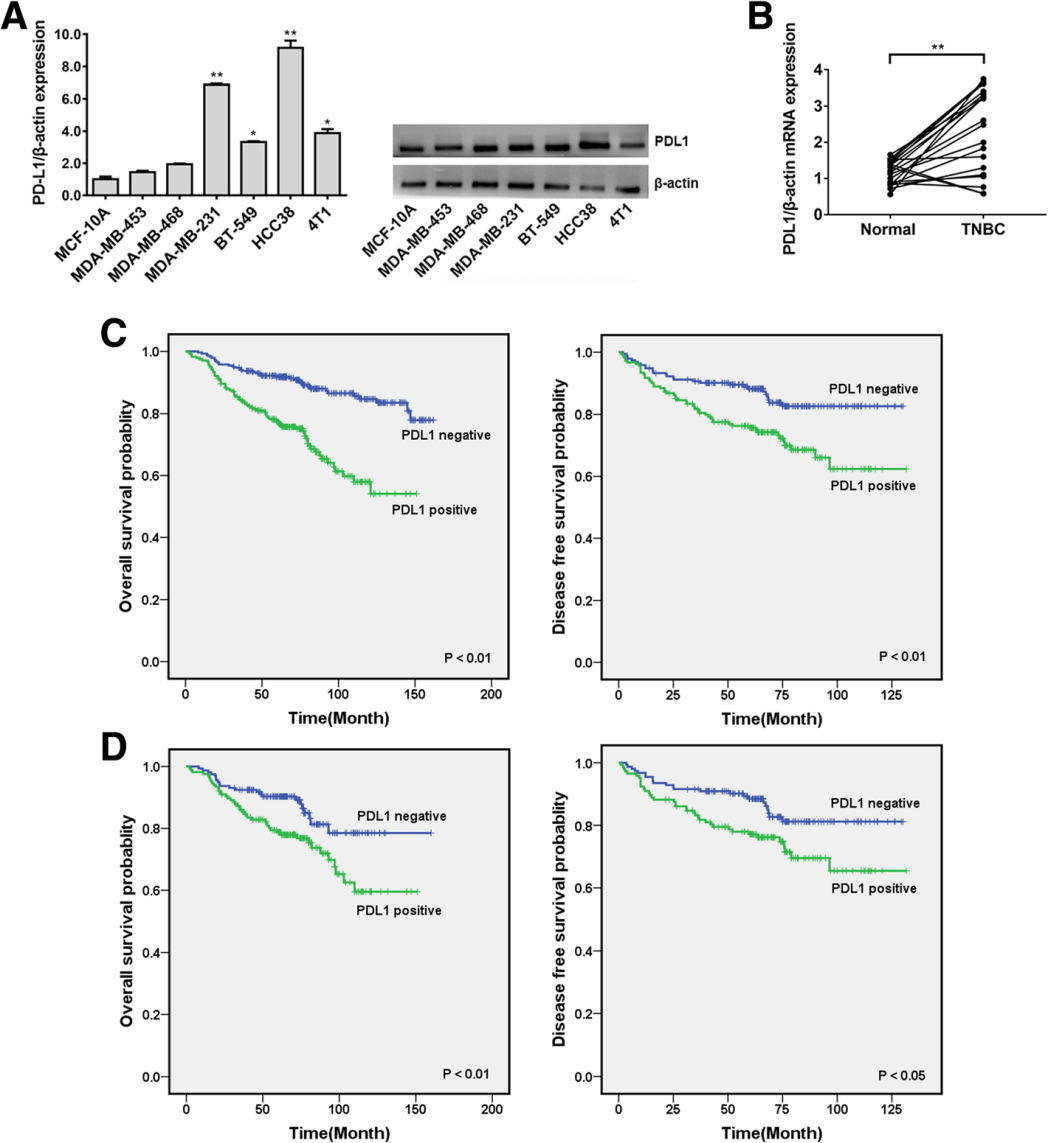
PDL1 is highly expressed in TNBC and correlated with a poor outcome. **a** The expression level of PDL1 was determined by qRT-PCR and Western blotting in seven different mammary cell lines, including one HME cell line (MCF-10A) and six TNBC cell lines. PDL1 expression was normalized using β-actin expression. **b** The expression level of PDL1 in 20 pairs of TNBC tissues and their matched normal adjacent tissues. **c** OS (left) and DFS (right) curves for breast cancer patients with positive or negative PDL1 expression. **d** OS (left) and DFS (right) curves for TNBC patients with positive or negative PDL1 expression. All the data are shown as the mean ± s.e.m. *P < 0.05, **P < 0.01

We used qRT-PCR analysis and Western blotting to detect the expression of PDL1 in seven different mammary cell lines, including the HME cell line MCF-10A and six TNBC cell lines. The results showed that compared with MCF-10A cells, PDL1 was upregulated in TNBC cell lines, especially in HCC38 and MDA-MB-231 cells (Fig. 1a). Therefore, we chose the HCC38 and MDA-MB-231 cell lines for further study. Then, we detected the expression of PDL1 in 20 pairs of TNBC tissues and their matched normal adjacent tissues. The results showed that approximately 85% (*P* < 0.01, 17 of 20 patients) of TNBC tissues exhibited an increase in PDL1 expression levels (Fig. 1b).

Then, we explored the potential prognosis implications of PDL1 expression in breast cancer. TMAs containing 554 tissues from breast cancer patients were used for IHC analysis. Characteristics of these patients are summarized in Table 1. The patients were divided into two groups based on their PDL1 expression levels and approximately 47.3% (262/554) of patients had positive expression of PDL1. To explore the significance of PDL1 in clinical prognosis, we used a Kaplan-Meier survival analysis to construct overall survival (OS) and disease-free survival (DFS) curves. The results showed that positive expression of PDL1 was correlated with worse OS and DFS (*P* < 0.01 for both OS and DFS; Fig. 1c). We further assessed the prognosis significance of PDL1 expression in the TNBC subgroups. And approximately 51.1% (166/325) of TNBC patients had positive expression of PDL1. The results showed that positive expression of PDL1 was correlated with worse OS and DFS in TNBC (*P* < 0.01 for OS, *P* < 0.05 for DFS; Fig. 1d).

**Table 1:** Patient characteristics

Variables	No. of patients (*n* = 554)	%
Age (years)		
<50	295	53.2
> = 50	259	46.8
Tumor size (cm)		
= < 2	143	25.9
>2	409	74.1
LNMET		
Yes	306	55.9
No	241	44.1
TNM stage		
I-II	350	63.5
III- IV	201	36.5
ER status		
Positive	155	30.0
Negative	362	70.0
PR status		
Positive	147	28.4
Negative	370	71.6
HER-2 status		
Positive	56	10.9
Negative	457	89.1
TNBC		
Yes	325	62.9
No	192	37.1

### PDL1 Is a target of miR-34a, and its functions could be inhibited by miR-34a

**Fig. 2:**
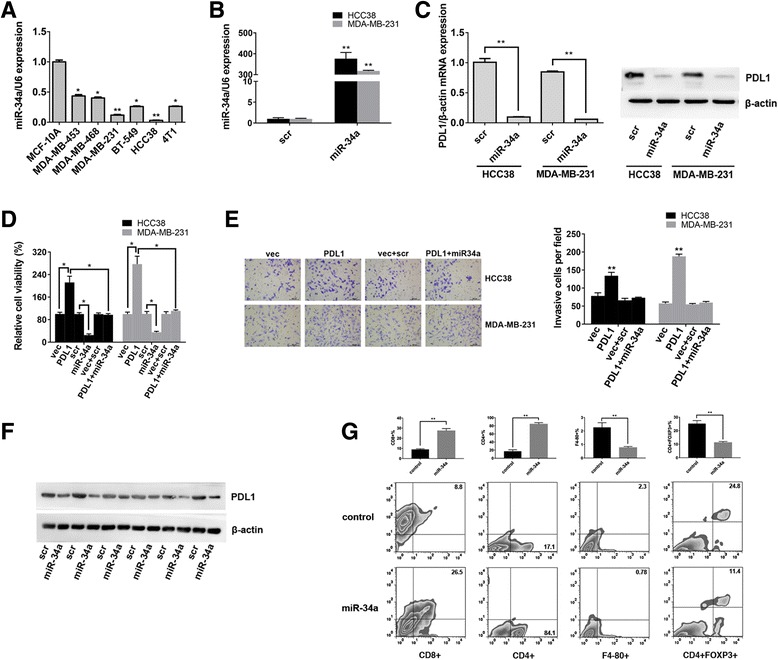
PDL1 is a target of miR-34a, and its functions could be inhibited by miR-34a. **a** The expression level of miR-34a was determined by qRT-PCR in the above cell lines. U6 snRNA was used as an internal control. **b** HCC38 and MDA-MB-231 cells were transfected with miR-34a mimic or scrambled oligonucleotide, and qRT-PCR analysis demonstrated that the transfection was successful. **c** HCC38 and MDA-MB-231 cells were transfected as described, and the mRNA and protein expression of PDL1 was suppressed by miR-34a. **d** Histogram presenting cell viability based on MTS assays for HCC38 and MDA-MB-231 cells 48 h after transfection. **e** Transwell invasion assays demonstrated that the PDL1 3’UTR promoted cell invasion. Representative images of invaded cells are shown in the left panel, and the results are summarized in the right panel. **f** The expression levels of PDL1 were determined by Western blotting in xenograft tumors (six in each group). β-Actin was used as an internal control. **g** The impact of miR-34a on immune cell populations in the tumor microenvironment. Flow cytometry revealed that miR-34a increased the number of CD8+ cells and CD4+ cells and reduced the number of macrophages and Tregs. All the data are shown as the mean ± s.e.m. *P < 0.05, **P < 0.01

Recently, it has been reported that PDL1 is a downstream target of miR-34a and that miR-34a directly targets the 3′ UTR of PDL1 [11, 12]. To further explore the correlation between PDL1 and miR-34a in TNBC, we detected the expression level of miR-34a in the above cell lines. The results showed that miR-34a was downregulated in TNBC cell lines (Fig. 2a). Then, we transfected HCC38 and MDA-MB-231 cells with a miR-34a mimic (Fig. 2b). Western blots and qRT-PCR analysis confirmed that the expression of PDL1 could be suppressed by miR-34a (Fig. 2c).

To examine the effect of the PDL1 3’UTR on proliferation, HCC38/PDL1 and MDA-MB-231/PDL1 cells were cultured, and MTS assays were performed. The results showed an obvious increase in cell viability after overexpression of the PDL1 3′ UTR (Fig. 2d). However, when the miR-34a mimic was delivered into cells, the cell viability significantly decreased (Fig. 2d).

Next, we explored the effect of the PDL1 3’UTR on cell invasion. HCC38/PDL1 and MDA-MB-231/PDL1 cells were cultured, and then Transwell assays were performed. The results showed that the PDL1 3′ UTR promoted cell invasion, and miR-34a could reverse the effect (Fig. 2e).

To further confirm the correlation between PDL1 and miR-34a in vivo, xenograft experiments were performed. Briefly, we inoculated MDA-MB-231 cells subcutaneously into nude mice. One week later, the mice were treated with miR-34a mimic or scrambled oligonucleotide (six mice in each group). After 28 days, the mice were euthanized, and the tumors were subjected to Western blotting. We found that ectopic expression of miR34a led to a significant decrease in the expression of PDL1 (Fig. 2f).

Then, we continued to explore the effects of miR-34a and the PDL1 3′ UTR on immune cell populations in the tumor microenvironment. Briefly, we inoculated 4 T1/PDL1 cells subcutaneously into immunocompetent C57BL/6 mice. One week later, the mice were treated with miR-34a mimic or scrambled oligonucleotide (five mice in each group). After 28 days, the mice were euthanized, and tumor lysates were subjected to flow cytometry. The results showed that miR-34a increased the number of tumor-infiltrating CD4^+^ cells and CD8^+^ cells and reduced the number of macrophages and Tregs (Fig. 2g).

These findings indicated that overexpression of the PDL1 3’UTR resulted in TNBC cell proliferation, invasion and an immunosuppressive tumor environment, and the above effect could be reversed by miR-34a.

### LDHA is highly expressed in TNBC and correlated with a poor outcome

**Fig. 3:**
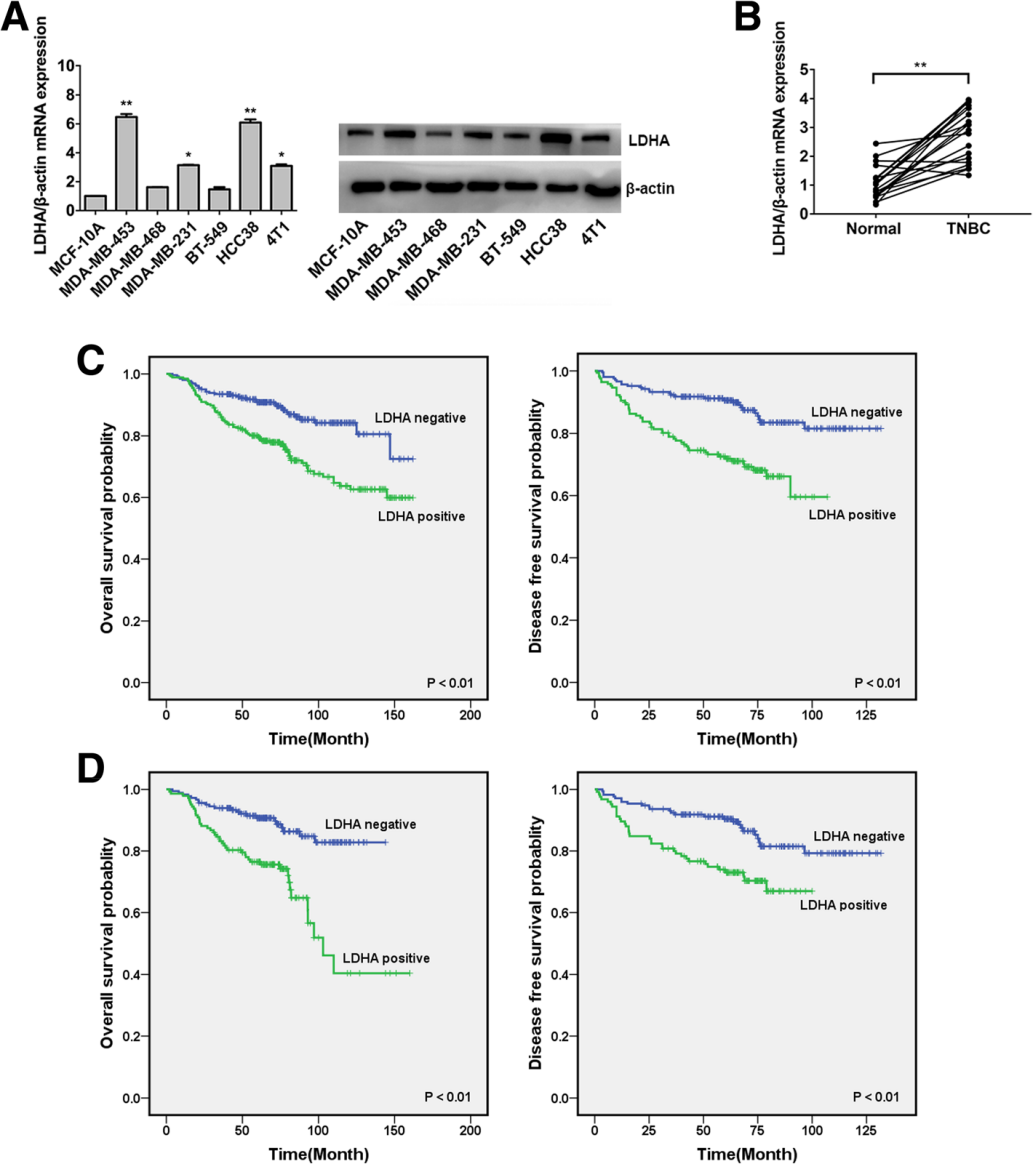
LDHA is highly expressed in TNBC and correlated with a poor outcome. **a** The expression level of LDHA was determined by qRT-PCR and Western blotting in the above cell lines. β-Actin was used as an internal control. **b** The expression levels of LDHA in 20 pairs of TNBC tissues and their matched normal adjacent tissues. **c** OS (left) and DFS (right) curves for breast cancer patients with positive or negative LDHA expression. **d** OS (left) and DFS (right) curves for TNBC patients with positive or negative LDHA expression. All the data are shown as the mean ± s.e.m. *P < 0.05, **P < 0.01

We detected the expression level of LDHA in the above cell lines. The results showed that LDHA was upregulated in TNBC cell lines compared with MCF-10A cells (Fig. 3a). Then, we detected the expression of LDHA in the above 20 pairs of tissues. Approximately 90% (*P* < 0.01, 18 of 20 patients) of the TNBC tissues showed an increase in LDHA expression (Fig. 3b).

Next, we explored the significance of LDHA in clinical prognosis using the above TMAs, and a Kaplan-Meier survival analysis was performed. The results showed that positive expression of LDHA was correlated with worse OS and DFS (*P* < 0.01 for both OS and DFS; Fig. 3c). In addition, positive expression of LDHA was also correlated with worse OS and DFS in TNBC tissues (*P* < 0.01 for both OS and DFS; Fig. 3d).

### Expression of PDL1 and LDHA is correlated and indicates a poor outcome in breast cancer

**Fig. 4:**
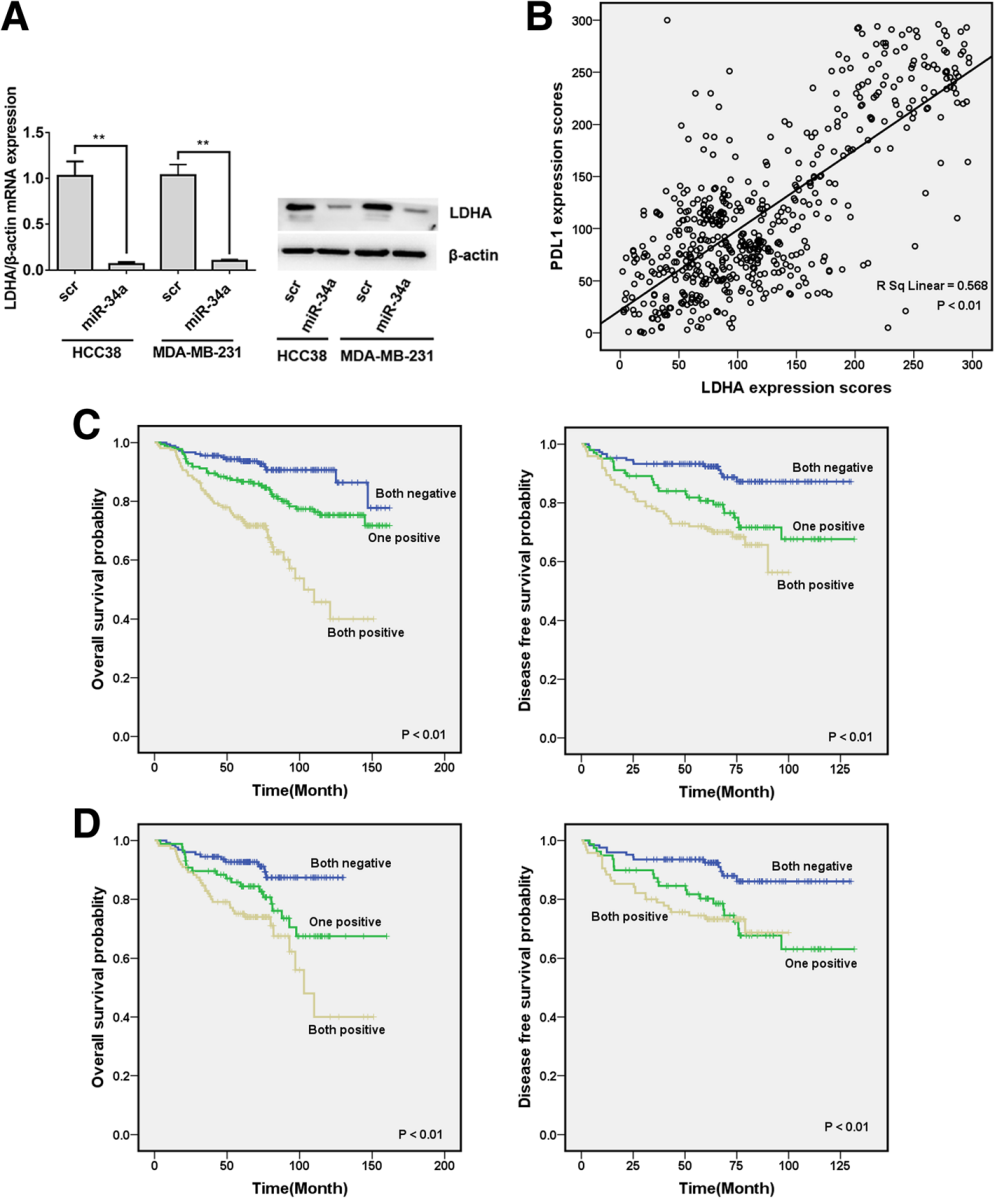
Expression of PDL1 and LDHA is correlated and indicates a poor outcome in breast cancer**. a** HCC38 and MDA-MB-231 cells were transfected with miR-34a mimic or scrambled oligonucleotide. The mRNA and protein expression of LDHA was suppressed by miR-34a. **b** The correlation between PDL1 and LDHA expression in 554 breast cancer patients was analyzed, and a positive correlation between them was found. **c** OS (left) and DFS (right) curves for breast cancer patients. **d** OS (left) and DFS (right) curves for TNBC patients. All the data are shown as the mean ± s.e.m. **P < 0.01

To further confirm that LDHA is also a target gene of miR-34a in TNBC, qRT-PCR and Western blot analyses were performed in HCC38 and MDA-MB-231 cells. The results showed a notable reduction in mRNA and protein levels of LDHA in cells infected with the miR-34a mimic (Fig. 4a). Then, we analyzed possible correlations between PDL1 and LDHA expression using the above TMAs and found that the expression of LDHA was positively correlated with that of PDL1 (Fig. 4b). To analyze the significance of PDL1 and LDHA in clinical prognosis of breast cancer patients, a Kaplan-Meier survival analysis was conducted. The results showed that patients with positive expression of both PDL1 and LDHA had shorter OS and DFS (*P* < 0.01 for both OS and DFS; Fig. 4c). We further performed a Kaplan-Meier survival analysis in the TNBC subgroups and found that patients in both positive groups also had shorter OS and DFS (*P* < 0.01 for both OS and DFS; Fig. 4d).

### PDL1 And LDHA act as ceRNAs in TNBC through regulation of miR-34a

**Fig. 5:**
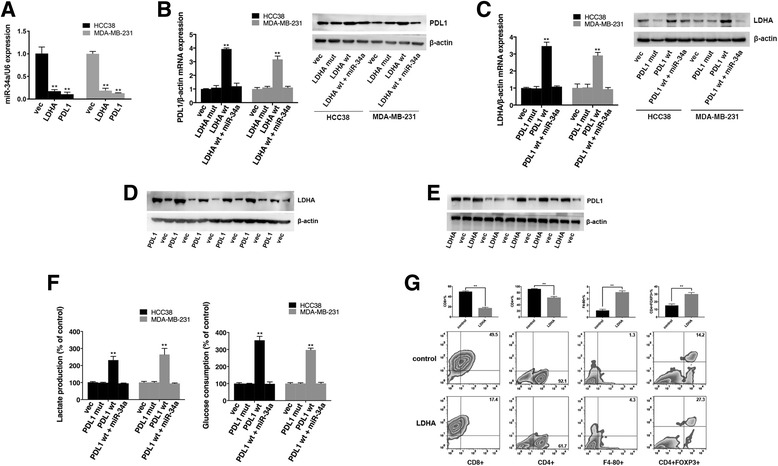
PDL1 and LDHA act as ceRNAs in TNBC by regulating miR-34a. **a** The expression level of miR-34a was determined by qRT-PCR in stable cell lines expressing the PDL1 3’UTR or LDHA 3’UTR. U6 snRNA was used as an internal control. **b** The expression level of PDL1 was determined by qRT-PCR and Western blotting. β-Actin was used as an internal control. **c** The expression level of LDHA was determined by qRT-PCR and Western blotting. β-Actin was used as an internal control. **d** The expression level of LDHA was determined by Western blotting in xenograft tumors (six in each group). β-Actin was used as an internal control. **e** The expression level of PDL1 was determined by Western blotting in xenograft tumors (six in each group). β-Actin was used as an internal control. **f** Lactate production and glucose consumption was evaluated by measuring the lactate and glucose levels in cell medium. **g** The impact of the LDHA 3’UTR on immune cell populations in the tumor microenvironment. Flow cytometry revealed that the LDHA 3’UTR increased the number of macrophages and Tregs and reduced the number of CD8+ cells and CD4+ cells. All of the data are shown as the mean ± s.e.m. **P < 0.01

Because the 3’UTRs of PDL1 and LDHA both have binding sites for miR-34a, we supposed that they could act as ceRNAs by competing for miR-34a. To confirm this supposition, we examined the expression levels of miR-34a in the stable cell lines expressing the PDL1 3’UTR or LDHA 3’UTR and found that the expression of miR-34a was suppressed (Fig. 5a). Overexpression of wild type LDHA 3’UTR resulted in increased levels of PDL1 while the mutant LDHA 3’UTR didn’t (Fig. 5b). Similarly, overexpression of wild type PDL1 3’UTR also increased the expression of LDHA (Fig. 5c). In addition, the above effect could be reversed by miR-34a (Fig. 5b and c). To further confirm the correlation between the PDL1 3’UTR and LDHA 3’UTR, xenograft experiments were performed. Briefly, we inoculated MDA-MB-231/PDL1 cells, MDA-MB-231/LDHA cells or MDA-MB-231/vec cells subcutaneously into nude mice. After 28 days, the mice were euthanized, and the tumors were subjected to Western blotting. We found that ectopic expression of the PDL1 3’UTR led to a significant increase in LDHA, and vice versa (Fig. 5d and e).

Because the PDL1 3’UTR increased the expression of LDHA, we assessed the biological effects of the PDL1 3’UTR on metabolic parameters. The results showed that enhanced expression of the PDL1 3’UTR led to increased lactate production and glucose consumption (Fig. 5f).

Then, we continued to explore the effects of the LDHA 3’UTR on immune cell populations in the tumor microenvironment. Briefly, we inoculated 4 T1/LDHA cells or 4 T1/vec cells subcutaneously into immunocompetent C57BL/6 mice (five mice in each group). After 28 days, the mice were euthanized, and tumor lysates were subjected to flow cytometry. The results showed that the LDHA 3’UTR increased the number of tumor-infiltrating macrophages and Tregs and reduced the number of CD4^+^ cells and CD8^+^ cells (Fig. 5g).

These results indicated that the PDL1 3’UTR and LDHA 3’UTR acted as ceRNAs by regulating miR-34a to promote the expression and function of each other.

## Discussion

Breast cancer is the most commonly diagnosed cancer in women worldwide. It is estimated that there will be 255,180 new cases and 41,070 deaths due to breast cancer in the United States in 2017 [16]. The last few decades have witnessed outstanding advances in breast cancer treatment. However, the prognosis for TNBC patients remains poor. Therefore, it is of great importance to develop more effective therapeutic strategies to treat breast cancer, especially TNBC.

PDL1 is well known to negatively regulate TILs, which results in an immunosuppressive environment in cancers. However, PDL1 also has important tumor-intrinsic signaling and survival effects. Azuma T et al. reported that PDL1 prevents tumor cell apoptosis [17]. Other studies have shown that PDL1 regulates cell growth, metastasis and autophagy [18, 19]. In this study, we found that PDL1 was highly expressed in TNBC and correlated with a poor outcome (Fig. 1). In addition, overexpression of the PDL1 3′ UTR promoted cell proliferation and invasion (Fig. 2). These results indicate that PDL1 could be a target in TNBC treatment.

Recently, PDL1 has been reported to be involved in the regulation of tumor glucose metabolism [20]. Chang CH et al. found that blocking PDL1 dampened tumor glycolysis by inhibiting mTOR activity and decreasing the expression of glycolysis enzymes [21]. In addition, binding of PD1 and PDL1 rewires the metabolic phenotype of activated T cells toward a naive-like state [22]. On the other hand, metabolic reprogramming enables tumor cells to restrict the availability of glucose to T cells, suppressing anti-tumor immunity [23]. Brand et al. reported a novel mechanism whereby lactic acid produced by highly glycolytic tumors overcomes immune surveillance by impairing activation of infiltrating cytotoxic CD8+ T and NK cells [24]. In addition, tumor metabolism can be therapeutically regulated to improve immune function in tumors [25]. These findings indicate that drugs that combine immunotherapy and metabolically targeted treatments are a potential strategy for antitumor therapy.

LDHA, which is essential for the conversion of pyruvate into lactate, is preferentially expressed in cancer cells and is correlated with poor survival. Xie H et al. demonstrated that LDHA is essential for cancer-initiating cell survival and proliferation [7]. Inhibition of LDHA has an antiproliferative effect on cancer cells, such as breast cancer and liver cancer cells [26, 27]. Reduction of LDHA reduced ATP levels and induced significant oxidative stress and cell death in lymphoma and pancreatic cancer cells [28]. In this study, we found that LDHA was highly expressed in TNBC and correlated with a poor outcome (Fig. 3). All these findings confirm that LDHA is a promising target for anticancer therapy [29, 30].

It has been reported that RNAs can act as ceRNAs to co-regulate each other by competing for shared microRNAs [8, 9]. Studies by several groups have illustrated that mRNAs, pseudogenes, long noncoding RNAs (lncRNAs) and circular RNAs (circRNAs) may all serve as ceRNAs [31]. Pseudogene PTENP1 could regulate cellular levels of PTEN and exert a growth-suppressive role [32]. BRAF pseudogene acts as a ceRNA and elevates BRAF expression and MAPK activation [33]. LncARSR promoted sunitinib resistance by competitively binding miR-34/miR-449 to facilitate AXL and c-MET expression [34]. Linc-RoR functions as a ceRNA to regulate the expression of OCT4, SOX2 and NANOG in embryonic stem cells [35]. Memczak et al. found that the circRNA CDR1as binds to miR-7 and impairs midbrain development [36]. In addition, expression of the 3′ UTR of mRNA alone is capable of eliciting ceRNA effects [10]. Jeyapalan Z et al. reported that the CD44 3′ UTR overexpressed in breast cancer cells interacts with endogenous miRNAs to arrest their mRNA-targeting function [37]. All these findings indicate that ceRNA-mediated microRNA sequestration may contribute to the regulation of cancer.

Recent studies have revealed that miR-34a suppresses PDL1 expression by directly targeting the 3′ UTR of PDL1 in lung cancer and acute myeloid leukemia [11, 12]. In this study, we further confirmed that PDL1 was a target of miR-34a, and its functions could be inhibited by miR-34a (Fig. 2). On the other hand, a microarray analysis indicated that LDHA is a potential target of miR-34a [38]. Further studies confirmed that miR-34a targets LDHA in colorectal cancer and breast cancer [13]. In this study, we also confirmed that LDHA was a target of miR-34a, and the expression of PDL1 and LDHA was positively correlated. In addition, co-expression of PDL1 and LDHA indicated a poor outcome in breast cancer (Fig. 4). Further experiments confirmed that by competing for miR-34a, the PDL1 3’UTR and LDHA 3’UTR acted as ceRNAs to promote the expression and function of each other in TNBC (Fig. 5). Thus, simultaneously targeting PDL1 and LDHA, which would combine immunotherapy and metabolically targeted treatments, would be a potential strategy for TNBC treatment.

## Conclusions

In summary, this study revealed that PDL1 and LDHA were synchronously upregulated and correlated with a poor outcome in TNBC. In addition, by competing for miR-34a, the PDL1 3’UTR and LDHA 3’UTR acted as ceRNAs to promote the expression and function of each other. Simultaneously targeting PDL1 and LDHA, which would combine immunotherapy and metabolically targeted treatments, would be a potential strategy to better treat TNBC.
